# Development and evaluation of a Markov model to predict changes in schistosomiasis prevalence in response to praziquantel treatment: a case study of *Schistosoma mansoni* in Uganda and Mali

**DOI:** 10.1186/s13071-016-1824-7

**Published:** 2016-10-12

**Authors:** Arminder Deol, Joanne P. Webster, Martin Walker, Maria-Gloria Basáñez, T. Déirdre Hollingsworth, Fiona M. Fleming, Antonio Montresor, Michael D. French

**Affiliations:** 1 Schistosomiasis Control Initiative, Department of Infectious Disease Epidemiology, School of Public Health, Faculty of Medicine (St Mary’s campus) Imperial College London, London, W2 1PG UK; 2 Department of Pathology and Pathogen Biology, Centre for Emerging, Endemic and Exotic Diseases, Royal Veterinary College, University of London, Herts, London, AL9 7TA UK; 3 London Centre for Neglected Tropical Disease Research, Department of Infectious Disease Epidemiology, School of Public Health, Faculty of Medicine (St Mary’s campus), Imperial College London, London, W2 1PG UK; 4 Department of Mathematics, University of Warwick, Coventry, CV4 7AL UK; 5 Neglected Tropical Disease Department, World Health Organization, Avenue Appia, 20, Geneva, Switzerland

**Keywords:** Schistosomiasis, Markov modelling, Transmission dynamics, Transition probabilities, Praziquantel, Prevalence, Intensity

## Abstract

**Background:**

Understanding whether schistosomiasis control programmes are on course to control morbidity and potentially switch towards elimination interventions would benefit from user-friendly quantitative tools that facilitate analysis of progress and highlight areas not responding to treatment. This study aimed to develop and evaluate such a tool using large datasets collected during Schistosomiasis Control Initiative-supported control programmes.

**Methods:**

A discrete-time Markov model was developed using transition probability matrices parameterized with control programme longitudinal data on *Schistosoma mansoni* obtained from Uganda and Mali. Four matrix variants (A-D) were used to compare different data types for parameterization: A-C from Uganda and D from Mali. Matrix A used data at baseline and year 1 of the control programme; B used year 1 and year 2; C used baseline and year 1 from selected districts, and D used baseline and year 1 Mali data. Model predictions were tested against 3 subsets of the Uganda dataset: dataset 1, the full 4-year longitudinal cohort; dataset 2, from districts not used to parameterize matrix C; dataset 3, cross-sectional data, and dataset 4, from Mali as an independent dataset.

**Results:**

The model parameterized using matrices A, B and D predicted similar infection dynamics (overall and when stratified by infection intensity). Matrices A-D successfully predicted prevalence in each follow-up year for low and high intensity categories in dataset 1 followed by dataset 2. Matrices A, B and D yielded similar and close matches to dataset 1 with marginal discrepancies when comparing model outputs against datasets 2 and 3. Matrix C produced more variable results, correctly estimating fewer data points.

**Conclusion:**

Model outputs closely matched observed values and were a useful predictor of the infection dynamics of *S. mansoni* when using longitudinal and cross-sectional data from Uganda. This also held when the model was tested with data from Mali. This was most apparent when modelling overall infection and in low and high infection intensity areas. Our results indicate the applicability of this Markov model approach as countries aim at reaching their control targets and potentially move towards the elimination of schistosomiasis.

**Electronic supplementary material:**

The online version of this article (doi:10.1186/s13071-016-1824-7) contains supplementary material, which is available to authorized users.

## Background

In recent years there has been a renewed focus on the control and possible elimination of certain neglected tropical diseases (NTDs) by the global health community. One of the NTDs with the greatest human health and socio-economic burden is schistosomiasis, estimated to infect over 238 million people [1] at a global cost of 3.3–4.5 million disability-adjusted life years (DALYs). Approximately 85 % of people infected with schistosomes reside in sub-Saharan Africa (SSA), with the disease potentially causing over 200,000 deaths per year [2, 3]. National-scale control programmes are now in place in many countries, using preventive chemotherapy (PC) by mass drug administration (MDA) with praziquantel (PZQ) [4].

The pharmaceutical company Merck KGaA has donated over 290 million tablets of PZQ to the World Health Organization (WHO) and has committed up to a further 250 million tablets per year from 2016 [5]. The tablets are distributed by the Ministries of Health of endemic countries, where in some, non-governmental organizations such as the Schistosomiasis Control Initiative (SCI) provide technical support and assistance (and in some cases purchasing and supplying additional PZQ) to these programmes [6, 7]. Since its establishment in 2002, SCI has helped to provide over 140 million treatments for schistosomiasis to at-risk children and adults in SSA and the Middle East [8]. As part of the monitoring and evaluation (M&E) component that runs alongside the treatment campaigns, SCI has contributed to the collection of rich longitudinal datasets from numerous countries on the impact of treatment on prevalence, intensity and morbidity. Many schistosomiasis control programmes have been running for several years, and have achieved their primary target of controlling schistosomiasis-related morbidity (where the aim of “control” is reducing prevalence of heavy infection to < 5 % across sentinel sites at 75 % national coverage [9]), whether from intestinal schistosomiasis (caused predominantly by *Schistosoma mansoni*) or from urogenital schistosomiasis (caused predominantly by *S. haematobium*) [10]. With this in mind, the WHO, alongside its global partners, has set the agenda for the next stage of control. The London Declaration on NTDs in January 2012 endorsed the ambitious targets set by the WHO for the control and elimination of many NTDs, including schistosomiasis, with the elimination ‘as a public health problem’ from most WHO regions and by selected African countries by 2020 (i.e. reducing prevalence of heavy infection < 1 % in all sentinel sites) [9, 11, 12]. In some local settings, interruption of transmission is also anticipated, thereby accelerating elimination of the disease [12].

The impact of a control programme is often measured by changes in the prevalence and/or the intensity of infection. Preventive chemotherapy by MDA with PZQ has been demonstrated to be, in general, highly effective in reducing both the prevalence and intensity of schistosome infection [13–15]. The development of a user-friendly quantitative tool that uses these impact measurements to inform programme managers as to whether their programme is on target to meet their goals would be invaluable in assisting with programme design and evaluation and in providing an early warning of potential transmission ‘hotspots’ or poor programme performance.

A Markov statistical model was developed to capture soil-transmitted helminth (STH) infection dynamics through rounds of MDA (with benzimidazoles), by Montresor and colleagues in 2013 [16, 17]. The authors demonstrated that their model successfully predicted changes in the prevalence of *Ascaris lumbricoides*, *Trichuris trichiura* and hookworm (consisting of the two species that infect humans: *Ancylostoma duodenale* and *Necator americanus*) through five rounds of MDA using data collected at baseline and after one round of treatment in Vietnam to parameterize the Markov Transition Probability (MTP) matrix; the essential ingredient of such Markov models. The predictive capability of the model was also successfully validated against STH data from 26 control programmes in 16 countries [17].

The main appeal of the Markov approach resides in its simplicity [18], whereby the underlying transmission dynamics are not modelled explicitly but are captured empirically using a purely statistical approach based on estimated transition probabilities (TP). The model can be used to track progress and to identify deviations from expected programme performance where observed values fall outside of predicted uncertainty intervals (e.g. 95 % prediction intervals, PIs).

Here, for the first time, we extend the discrete-time Markov model approach, in which both time and infection states (intensity groups) are defined, and apply it to *S. mansoni*, a causative agent of intestinal schistosomiasis across Africa, South America, and the Yemen. We test the model under contrasting control programme scenarios, using unique and extensive datasets from SCI-supported programmes in Uganda and Mali.

Our specific aims in this study were to: (i) develop and test a discrete-time Markov model for schistosomiasis using data on the intensity and prevalence of *S. mansoni* infection during mass treatment with PZQ; (ii) introduce measurements of precision around predictions in the form of 95 % PIs; (iii) estimate changes in the overall infection prevalence and the prevalence in infection intensity categories over time; (iv) qualitatively compare the predictive capabilities of the model parameterized using MTP matrices estimated from different settings within the same country (Uganda) and from a different country (Mali), to test the transferability of the TPs to different regions; (v) test the robustness of the model’s predictive capabilities using data from non-baseline years to estimate the MTP matrices; and (vi) elucidate the ability of different data types (longitudinal and cross-sectional data) to qualitatively test the predictions of each matrix.

## Methods

### Model development

**Fig. 1:**
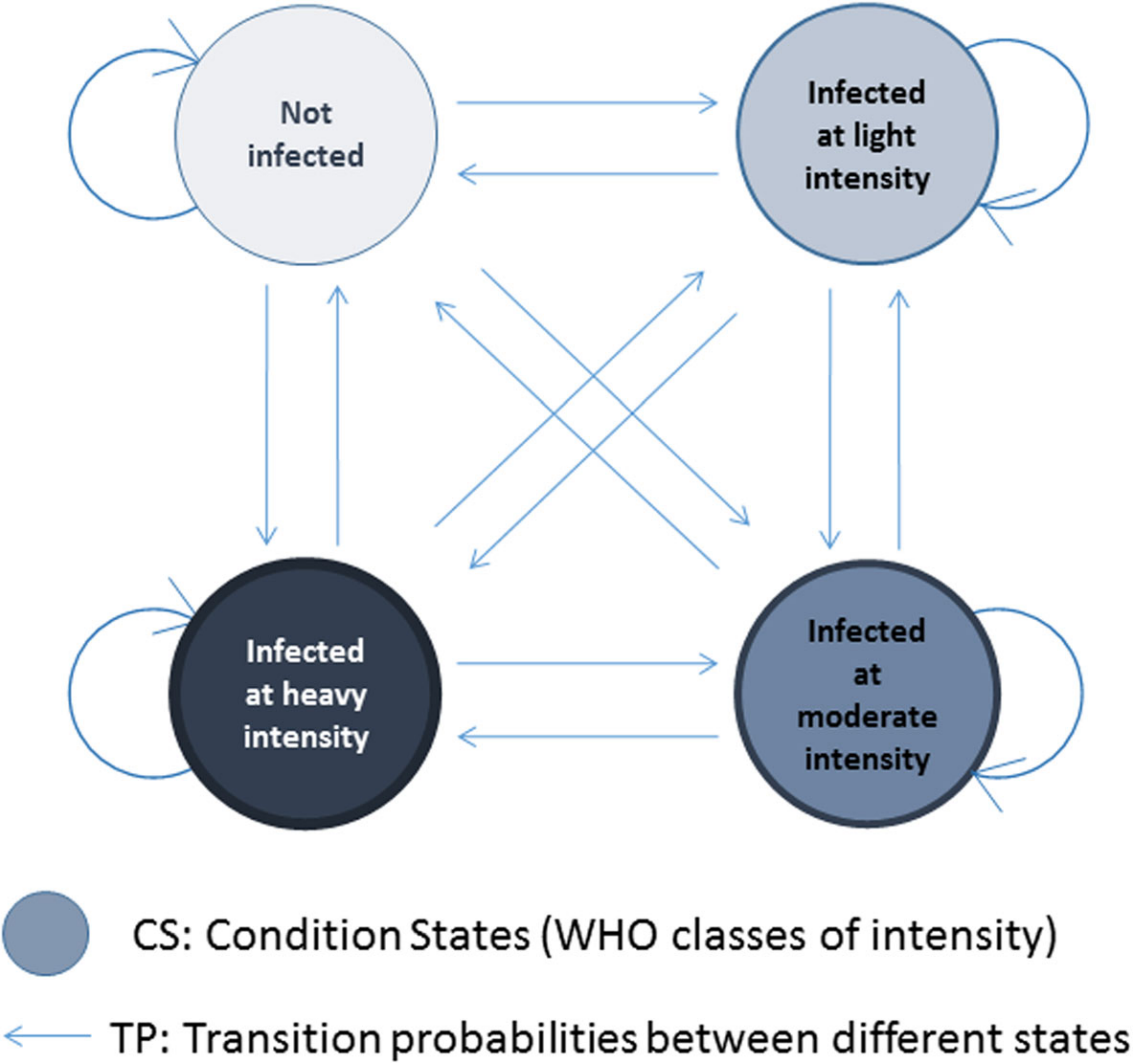
Transition diagram illustrating a Markov transition probability matrix [16]

The development of a Markov model for STH infection has been explained fully elsewhere [16]. Briefly, in relation to *S. mansoni,* the proportion of individuals in each of the 4 WHO-recommended infection classes defined by estimates of eggs per gram (epg) of faeces (not infected, 0 epg; infected at light intensity, 1–99 epg; infected at moderate intensity, 100–399 epg; infected at high intensity, ≥ 400 epg [19]) and referred to as “conditional states” (CS), is calculated from pre-treatment baseline data [20]. Subsequently, an individual’s probability of transition (if any) to other CS prior to the next round of treatment (year 1) is calculated using the observed change in the proportion of followed individuals in each category (from baseline to year 1). These observed changes are used to parameterize a MTP matrix, formed from a set of 16 transition probabilities (TPs), as illustrated in Fig. 1. The model is initialised using observed baseline starting values. Then, through a series of Markov processes defined by the MTP matrix (see Additional file 1: Text S1 and Table S1), projections are made on the proportion of infected individuals by intensity class through rounds of MDA.

In the first instance, we focused on *S. mansoni* data collected from Uganda between the inception of the programme in 2003 and for the first 3 annual follow-up rounds after baseline. For further details of the control programme in Uganda see [21, 22]. As part of the national control programme, data were collected as egg counts (expressed as the arithmetic mean epg) from a cohort of 125 children (aged 6–13) per school, from 37 schools across the country, over a time span of 4 years.

For the calculation of the TPs from the full Uganda dataset, longitudinal data between baseline and year 1 were used (i.e. only data from those individuals who could be identified at each of those time points, namely 1,258 individuals). To quantify uncertainty around the model projections (expanding on the previously published version of the model applied to STH [15, 16]), 95 % prediction intervals (95 % PIs) associated with each TP were calculated through bootstrap resampling (with replacement) for 10,000 iterations, using the R package ‘boot’ version 1.3–9 [23–26]. The 95 % PIs were calculated in the following steps: 1) a new ‘dataset’ was generated through bootstrapping allowing for the calculation of a new MTP matrix (set of 16 TPs); 2) the model was run (using these TPs) to calculate the reduction in prevalence over time; 3) steps 1) and 2) were repeated 10,000 times; 4) for each time point, the predicted mean prevalence was calculated; and 5) from the range of predicted prevalence levels generated, the 95 % PIs were constructed using the 2.5 % and 97.5 % percentiles. Initially, for the observed data, the full cohort of individuals who were followed up from baseline to year 3 of the intervention was included (757 individuals). Since some of the individuals in this dataset were also used for the calculation of the TPs (as would be the case in practice when using these models), it was expected that the predicted prevalence at year 1 would follow the observed values from the full dataset 1 (Table 1) very closely. In order to test the transferability of the model using independent data, the TPs calculated from the full Uganda dataset were also used to test model predictions against longitudinal data from Mali. Conversely, to further test the robustness of the model, longitudinal baseline and year 1 data from Mali was also used to parameterize a separate model and tested against observed Uganda longitudinal data. These additionally tested the flexibility of the model to different starting baseline prevalence levels (for Mali the baseline overall prevalence was 26.5 % for *S. mansoni* infection whilst for Uganda the overall prevalence was 43.0 %).

**Table 1:** Data used for testing model/matrices

Observed baseline prevalence (%)							
Dataset	Data type	Description	Sample size (*n*)	Overall prevalence	Low intensity	Moderate intensity	High intensity
1	Uganda longitudinal baseline to year 3	Full longitudinal data set	747	43.0	16.6	11.4	15.0
2	Uganda longitudinal baseline to year 3	4^a^ Ugandan districts out of 7	400	46.5	15.5	12.3	18.8
3	Uganda cross-sectional baseline to year 3	Varying sample size per year, full programme data	Baseline: 4,222; Year 1: 3,973; Year 2: 4,192; Year 3: 3,373	45.2	16.0	11.7	17.6
4	Mali longitudinal baseline to year 2	Full longitudinal data set	897	26.5	12.5	7.1	6.9

^a^These districts were selected for their wide range of infection intensities and NOT used to the development of matrix C

### Datasets used and models developed

**Fig. 2:**
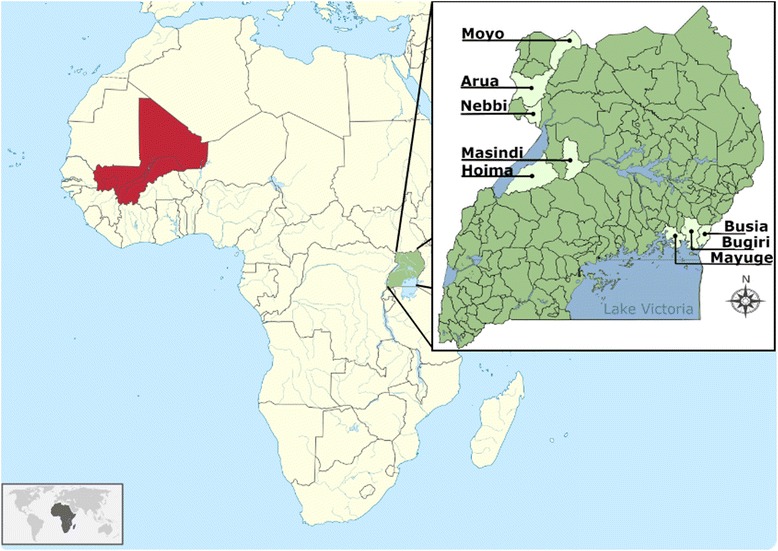
Map of Africa showing Mali (*red*) and Uganda (*green*). Subset: Uganda by district in study sample

The data were collected as part of a treatment campaign in Uganda for school-aged children (SAC) from 2003 to 2006 and in Mali from 2004 to 2007 (Fig. 2). We selected SCI data from Uganda as our primary dataset to parameterize and validate our model because: (i) Uganda was the first ‘SCI country’ to commence large-scale control of schistosomiasis in 2003, and thus has the most extensive longitudinal datasets (including pre-intervention baseline); (ii) *S. haematobium* infections are highly localised to specific regions within Uganda, with prevalence mostly below 1 %, and hence the potentially confounding impact of *S. haematobium* infection on the transition probabilities can be assumed to be minimal [27]; and (iii) Uganda has been very successful in implementing control [13], making this country an ideal candidate to move towards elimination of schistosomiasis as a public health problem. The extensive Ugandan dataset also enabled the model to be tested against data obtained from contrasting districts and disease endemicities. Three districts were selected based on their geographic spread and the distribution of infection intensities: Moyo (only low intensity infections); Busia (only low and moderate intensity infections); Masindi (only moderate and high intensity infections). There were no districts with only moderate or only high infection intensities. The remaining districts on which the model was tested (i.e. dataset 2) contained a varied composition of intensities (and were *not* used for the development of matrix C) (see Fig. 2 and Additional file 1: Table S2 for further details on the districts). The dataset and its different subsets that were used to test the predictive capabilities of the models are listed in Table 1. Table 2 shows other MTP matrices that were developed by the same method described in the previous sub-section, *Model development*.

**Table 2:** Markov transition probability (MTP) matrices developed

MTP matrix	Country	Number of districts	Time points used to develop matrix	Sample size (*n*)
A	Uganda	7	Baseline and year 1	1,245
B	Uganda	7	Year 1 and year 2	1,260
C	Uganda	3	Baseline and year 1	540
D	Mali	-	Baseline and year 1	1,092

In summary, 4 matrix variants (A-D) were used to compare different data types for parameterization: A-C from Uganda and D from Mali. These were tested on 4 datasets (1–4): dataset 1 refers to the full longitudinal cohort data from Uganda; dataset 2 to a subset of dataset 1 using districts not used to parameterize matrix C; dataset 3 to cross-sectional data from Uganda, and dataset 4 comprises data from Mali, which acted as a completely independent dataset. Matrix A was an ‘ideal’ scenario where longitudinal baseline and year 1 data from a large programme were available to parameterize the model and develop the TPs. The TPs were assumed to be fixed throughout the years. In practice, since changes between intensity groups are likely to be more dramatic after the first treatment in a treatment-naïve area, matrix B was developed using TPs from post-baseline treatment, between year 1 and year 2. The use of matrix C predictions on dataset 2 is an illustration of a scenario where an ‘independent’ matrix might be used, calculated from a smaller dataset, to estimate changes on a ‘separate’ smaller dataset (dataset 2) that is not used to develop the TPs. Matrix D illustrates a case where longitudinal data from another country are used to develop the TPs (Mali) in order to predict changes in prevalence in a separate country (Uganda). In the following sections we distinguish between ‘estimation’ (the estimated TP values), ‘prediction’ (the model outputs), 95 % prediction intervals (95 % PIs, constructed as described above) and 95 % confidence intervals (95 % CIs) around the data (calculated as binomial proportion confidence intervals). As a conservative approach to the qualitative model assessment, we focus on the ability of the models to capture the observed point prevalence values within the 95 % PIs whilst also highlighting whether the 95 % PIs of the model capture the 95 % CIs of the observed data.

### Matrix and dataset combinations

#### Matrix A, datasets 1, 2, 3, 4

Matrix A was calculated using all 1,245 individuals that were followed from baseline to year one in the Uganda dataset. Dataset 1 contains 747 of these individuals who were followed for a further 3 years (lower numbers due to loss of follow-up). Therefore, we expected Matrix A to provide the most accurate predictions, on dataset 1. In addition, to test how the model performed with smaller sample sizes, less complete data, and other data types, selected districts (dataset 2) and cross-sectional data (dataset 3) were used. To test how well the model performed using matrix A on a completely independent dataset, longitudinal data from Mali (baseline to year 2; dataset 4) were used.

#### Matrix B, datasets 1, 2, 3

It is important to understand how the model and its outputs differ between 2 different time points within the same settings, since the model explicitly assumes that the TPs remain constant between each time point. To explore this, instead of using the baseline and year 1 data to calculate the TPs for the matrix, data derived from follow-up years 1 and 2 were used from the full Uganda dataset (matrix B). The outputs from these TPs were compared to the observed values from datasets 1–3.

#### Matrix C, datasets 1, 2, 3

A comparison was made between model outputs using smaller sample sizes for situations in which fewer data are available to parameterize TPs. This was achieved by selecting district-level subsets of the data for calculating TPs. The predictions were also tested against dataset 1 (longitudinal Uganda dataset) to represent a case where limited data would be used for the development of the TPs to project the expected impact of a much larger programme. In addition, to test the least favourable data scenario where there is very high loss to follow-up, the model was also used to estimate changes in the proportions infected according to cross-sectional data, i.e. small sample size for TP development and poor follow-up to test the model (dataset 3).

#### Matrix D, dataset 1

Transition probabilities developed from the Mali baseline and year 1 data (Matrix D) were used to predict the longitudinal Ugandan dataset (dataset 1). This was performed by way of testing model performance when a dataset other than the Ugandan data are used for calculation of the TPs. This addresses issues on the generalizability of the MTP approach among endemic settings.

## Results

We focus on the ability of the models to capture the observed point prevalence values (and accompanying uncertainty) within the 95 % PIs. Where the upper or lower bounds of the 95 % CIs around the observed values overlapped with the model predictions (or their 95 % PIs) only, the model was able to capture the uncertainty in the data but not the point prevalence.

### Predictions made on dataset 1

Table 3 shows all the predictions that were made for dataset 1. The symbol ɤ next to the values highlights predictions that were closest to the observed point prevalence values and the values in bold highlight predictions where observed point prevalence values fell outside the 95 % PIs; in most cases however, the model still captured some of the uncertainty around the observed values (10 cases out of 13 shown in bold).

**Table 3:** Predicted mean prevalence by matrices A-D for dataset 1 (full Uganda cohort baseline year 0 – year 3)

	Low intensity (predicted mean prevalence and 95 % CI)			Moderate intensity (predicted mean prevalence and 95 % CI)			High intensity (predicted mean prevalence and 95 % CI)			Overall prevalence (predicted mean prevalence and 95 % CI)		
Matrix	Year 1	Year 2	Year 3	Year 1	Year 2	Year 3	Year 1	Year 2	Year 3	Year 1	Year 2	Year 3
**Observed prevalence dataset 1**	0.134(0.111–0.160)	0.099 (0.080–0.123)	0.102 (0.082–0.126)	0.075 (0.058–0.096)	0.021 (0.013–0.035)	0.033 (0.023–0.049)	0.035 (0.024–0.051)	0.016 (0.009–0.028)	0.020 (0.011–0.031)	0.244 (0.214–0.276)	0.137 (0.114–0.163)	0.154 (0.130–0.182)
**Matrix A** Full dataset	0.142 (0.123*–*0.161)	0.108 (0.091*–*0.126)	0.095^a^ (0.077*–*0.113)	0.075^a^ (0.062*–*0.090)	**0.044 (0.033** ***–*** **0.056)**	0.033^a^ (0.023*–*0.045)	0.044 (0.033*–*0.055)	0.023 (0.015*–*0.032)	0.017^a^ (0.010*–*0.026)	0.261 (0.240*–*0.282)	**0.175** **(0.151** ***–*** **0.200)**	0.144^a^ (0.119*–*0.171)
**Matrix B** Uganda year 1 to year 2	0.135^a^ (0.112*–*0.158)	0.105 (0.086*–*0.126)	0.090 (0.072*–*0.109)	0.069 (0.051*–*0.090)	**0.039 (0.028** ***–*** **0.051)**	0.028 (0.019*–*0.038)	0.048 (0.031*–*0.066)	0.024 (0.015*–*0.036)	0.016 (0.009*–*0.024)	0.252^a^ (0.225*–*0.278)	**0.168 (0.141** ***–*** **0.197)**	0.133 (0.108*–*0.160)
**Matrix C** 3 selected districts	0.152 (0.122*–*0.183)	0.096^a^ (0.071*–*0.122)	0.082 (0.057*–*0.108)	**0.045 (0.027** ***–*** **0.065)**	0.016^a^ (0.008*–*0.027)	**0.009 (0.003** ***–*** **0.017)**	0.027 (0.013*–*0.043)	0.011^a^ (0.003*–*0.021)	**0.008 (0.001** ***–*** **0.018)**	0.223 (0.193*–*0.255)	0.123^a^ (0.093*–*0.156)	**0.099 (0.069** ***–*** **0.132)**
**Matrix D** Mali full dataset	**0.165 (0.141** ***–*** **0.190)**	**0.122 (0.100** ***–*** **0.146)**	0.095^a^ (0.073*–* 0.117)	0.081 (0.062*–*0.101)	**0.051 (0.037** ***–*** **0.068)**	0.035 (0.023*–*0.049)	0.042^a^ (0.028*–*0.057)	0.021^a^ (0.012*–*0.032)	0.031 (0.007*–*0.021)	**0.288 (0.264** ***–*** **0.312)**	**0.195 (0.164** ***–*** **0.226)**	0.143 (0.113*–*0.175)

**Bold** = observed point prevalence values fell outside of the predicted boundaries

^a^Closest predictions to observed values

**Fig. 3:**
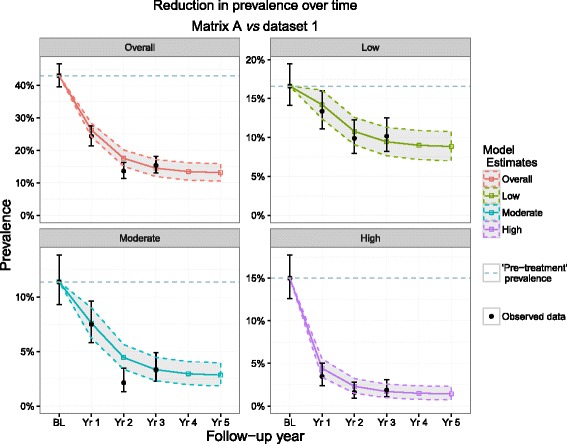
Matrix A predictions and dataset 1 observations. Matrix A was composed of transition probabilities derived from Uganda baseline and year 1 data and dataset 1 represents the full longitudinal Ugandan observations. These 4 plots show the predicted reduction in prevalence by Matrix A (bands) *vs* observed (black points) in Uganda by overall prevalence group and by intensity group. The dotted line represents the pre-MDA prevalence

All of the predictions from each matrix captured the observed point prevalence values within their PIs for the low infection intensity prevalence category in each year with the exception of matrix D (year 1 and marginally for year 2) and for the prevalence of high intensity infections with the exception of matrix C (year 3), although in both cases the 95 % PIs and the 95 % CIs overlapped. When using the TPs derived from matrix A (the full Ugandan dataset) to predict forward the reduction in *overall* infection prevalence as well as in prevalence for all infection intensity groups, the outputs matched the observed data within the 95 % PIs for all time points with the exception of the moderate intensity group and the overall prevalence for year 2 (Fig. 3 and Table 3), which indicated that the observed prevalence for each infection intensity group was below the lower bound of the prediction intervals of the estimated prevalence. However, in both instances, the model captured the 95 % CIs of the observed values.

**Fig. 4:**
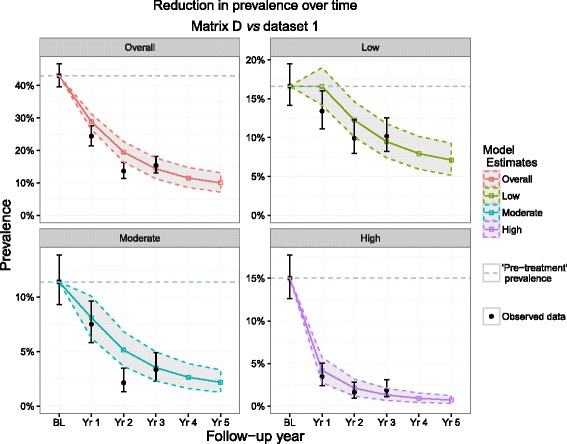
Matrix D predictions and dataset 1 observations. Matrix D was composed of transition probabilities derived from Mali baseline and year 1 data and dataset 1 represents the full longitudinal Ugandan observations. These 4 plots show the predicted reduction in prevalence by Matrix D (bands) *vs* observed (black points) in Uganda by overall prevalence group and by intensity group. The dotted line represents the pre-MDA prevalence

As with matrix A, matrices B (Additional file 1: Figure S1a) and D (Fig. 4) also ‘highlighted’ year 2 for both prevalence of moderate infection intensity and overall prevalence as a year in which observed values fell below 95 % PIs (with matrix B capturing the upper 95 % CI around the data, as with matrix A). Matrix C, however, did not highlight any of the same time points identified by the other matrices but instead, highlighted different years in the moderate intensity, high intensity and overall prevalence groups as time points in which observed point prevalence levels were higher than predicted by the model (Additional file 1: Figure S1b).

### Predictions made on dataset 2

Table 4 shows the predictions that were made for dataset 2 (see also Additional File 1: Figure S2). All 3 matrices in this group indicated the same time point for the low infection intensity group (year 3) and the overall prevalence group (year 1 and year 3) as performing below the expected values, i.e. higher observed point prevalence values than predicted (although matrix A also identified year 2 for better programme performance than expected, for overall infection prevalence). The same pattern in predicted *vs* observed prevalence from dataset 1 by all matrices was observed in the moderate infection intensity group for all time points, with the exception of year 3 for matrix B, which mirrored matrix C estimates. Matrices A and B performed similarly as in dataset 1 for the high intensity group (i.e. all observations at each time point were within the prediction intervals of the model predictions) but matrix C indicated that the observed prevalence values from years 1 and 2 were marginally higher than expected. Matrix A was able to capture the uncertainty in all 12 observed values of dataset 2, matrix B captured 10 out of 12 and matrix C captured 9 out 12.

**Table 4:** Predicted mean prevalence by matrices A-C for dataset 2 (selected Ugandan districts)

	Low intensity (predicted mean prevalence and 95 % CI)			Moderate intensity (predicted mean prevalence and 95 % CI)			High intensity (predicted mean prevalence and 95 % CI)			Overall prevalence (predicted mean prevalence and 95 % CI)		
Matrix	Year 1	Year 2	Year 3	Year 1	Year 2	Year 3	Year 1	Year 2	Year 3	Year 1	Year 2	Year 3
**Observed prevalence dataset 2**	0.158 (0.125–0.196)	0.105 (0.079–0.139)	0.143 (0.112–0.180)	0.100 (0.074–0.133)	0.020 (0.010–0.030)	0.045 (0.029–0.070)	0.055 (0.037–0.082)	0.030 (0.017–0.052)	0.018 (0.009–0.036)	0.313 (0.269–0.360)	0.155 (0.123–0.194)	0.205 (0.168–0.247)
**Matrix A** Full dataset	0.152^a^ (0.133–0.172)	0.112 (0.095–0.130)	**0.096** **(0.078–0.115)**	0.085^a^ (0.070–0.101)	**0.048 (0.036–0.060)**	0.034^a^ (0.024–0.046)	0.051 (0.039–0.063)	0.025 (0.017–0.035)	0.018^a^ (0.010–0.026)	**0.289 (0.268–0.311)**	**0.185 (0.161–0.211)**	**0.148** **(0.123–0.175)**
**Matrix B** Uganda year 1 to year 2	0.140 (0.115–0.166)	0.109^a^ (0.089–0.129)	**0.092** **(0.074–0.111)**	0.078 (0.055–0.102)	**0.042 (0.030–0.055)**	**0.029** **(0.020–0.039)**	0.055^a^ (0.035–0.077)	0.027^a^ (0.016–0.040)	0.017 (0.009–0.026)	**0.272 (0.242–0.302)**	0.178^a^ (0.149–0.208)	**0.137** **(0.111–0.165)**
**Matrix C** 3 selected districts	0.166 (0.132–0.199)	0.099 (0.075–0.124)	**0.082** **(0.057–0.108)**	**0.052 (0.031–0.075)**	0.018^a^ (0.009–0.029)	**0.010** **(0.003–0.018)**	**0.031 (0.014–0.051)**	**0.012 (0.003–0.023)**	0.008 (0.001–0.018)	**0.249 (0.216–0.282)**	0.129 (0.098–0.162)	**0.100 (0.070–0.132)**

**Bold** = observed point prevalence values fell outside of the predicted boundaries

^a^ Closest predictions to observed values

### Predictions made on dataset 3

**Fig. 5:**
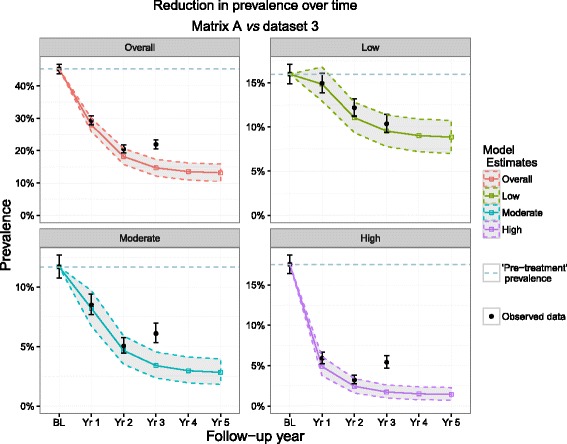
Matrix A (full Ugandan baseline and year 1 transition probabilities) predictions and dataset 3. Dataset 3 represents cross-sectional Uganda observations. These 4 plots show the predicted reduction in prevalence by Matrix A (bands) *vs* cross-sectional observed (black points) in Uganda by overall prevalence group and by intensity group. The dotted line represents the pre-MDA prevalence

Table 5 shows the predictions that were made for dataset 3 (cross-sectional observed data). Figure 5 shows the output obtained from using the matrix A model on dataset 3 and Additional File 1: Figure S3 shows the plots corresponding to applying matrices B and C on dataset 3.

**Table 5:** Predicted mean prevalence by matrices A-C for dataset 3 (cross-sectional Ugandan data)

	Low intensity (predicted mean prevalence and 95 % CI)			Moderate intensity (predicted mean prevalence and 95 % CI)			High intensity (predicted mean prevalence and 95 % CI)			Overall prevalence (predicted mean prevalence and 95 % CI)		
Matrix	Year 1	Year 2	Year 3	Year 1	Year 2	Year 3	Year 1	Year 2	Year 3	Year 1	Year 2	Year 3
**Observed prevalence dataset 3**	0.150 (0.139–0.161)	0.122 (0.112–0.132)	0.104 (0.094–0.115)	0.085 (0.077–0.094)	0.051 (0.044–0.053)	0.061 (0.053–0.070)	0.059 (0.052–0.070)	0.032 (0.028–0.038)	0.054 (0.048–0.062)	0.294 (0.280–0.308)	0.205 (0.193–0.218)	0.219 (0.205–0.233)
**Matrix A** Full dataset	0.149^a^ (0.130*–*0.168)	0.111^a^ (0.093*–*0.128)	0.095^a^ (0.078*–*0.114)	0.082^a^ (0.068*–*0.097)	0.047^a^ (0.035*–*0.059)	**0.034 (0.024** ***–*** **0.045)**	0.049 (0.037*–*0.061)	0.024 (0.016*–*0.034)	**0.017** **(0.010** ***–*** **0.026)**	0.280^a^ (0.259*–*0.301)	0.182^a^ (0.157*–*0.207)	**0.147** **(0.121** ***–*** **0.173)**
**Matrix B** Uganda year 1 to year 2	0138 (0.114*–*0.163)	0.108 (0.088*–*0.128)	0.091 (0.073*–*0.110)	0.075 (0.053*–*0.098)	0.041 (0.029*–*0.054)	**0.028** **(0.019** ***–*** **0.039)**	0.052^a^ (0.033*–*0.073)	0.026^a^ (0.016*–*0.039)	**0.017** **(0.009** ***–*** **0.025)**	0.265 (0.235*–*0.295)	0.174 (0.146*–*0.205)	**0.136** **(0.110** ***–*** **0.163)**
**Matrix C** 3 selected districts	0.160 (0.128*–*0.193)	0.098 (0.074*–*0.123)	0.082 (0.057*–*0.108)	**0.050 (0.029** ***–*** **0.072)**	**0.017 (0.008** ***–*** **0.029)**	**0.009** **(0.003** ***–*** **0.018)**	**0.030 (0.014** ***–*** **0.049)**	**0.011 (0.003** ***–*** **0.022)**	**0.008** **(0.001** ***–*** **0.018)**	**0.240 (0.208** ***–*** **0.273)**	**0.127 (0.096** ***–*** **0.159)**	**0.100** **(0.070** ***–*** **0.131)**

**Bold** = observed point prevalence values fell outside of the predicted boundaries

^a^ Closest predictions to observed values

All data points in the low intensity of infection prevalence group were estimated accurately by each matrix, where both the observed point prevalence values as well as their 95 % CIs were captured by the model. As with dataset 1, matrices A and B produced similar outputs, with the observed data points and their 95 % CIs predicted by the models, with the exception of year 3, in moderate intensity, high intensity and overall prevalence groups. For matrix C, other than the low infection intensity group, the observed prevalence levels in all of the other infection intensity groups in all years were greater than the predicted range.

### Predictions made on dataset 4

**Fig. 6:**
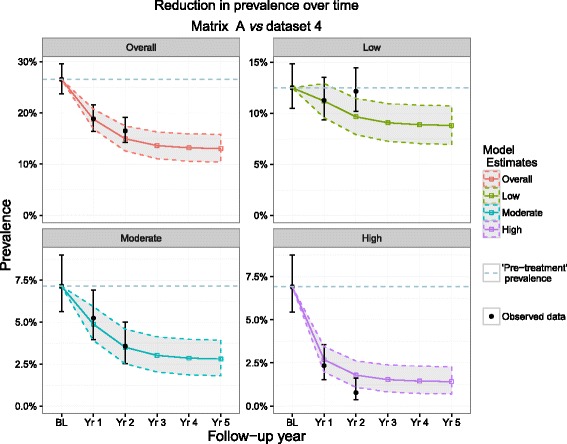
Matrix A (Uganda baseline and year 1 transition probabilities) predictions and dataset 4. Dataset 4 represents full longitudinal Mali observations. These 4 plots show the predicted reduction in prevalence by Matrix A (bands) *vs* observed (black points) in Mali by overall prevalence group and by intensity group. The dotted line represents the pre-MDA prevalence

Figure 6 and Table 6 show the model outputs when Ugandan TPs were used to estimate changes in the longitudinal data from Mali. The results show that the model predictions match the changes in prevalence closely, with only year 2 observations from the low and high infection intensity groups falling outside of the prediction intervals, yet capturing the uncertainty around the data. The low intensity year 2 prediction shows an increase in prevalence, but inspection of the high intensity group shows that this may be due to individuals moving from the higher infection intensity groups to the low intensity and the non-infected group. Additional File 1: Figure S4 also shows the output obtained when applying Matrix D to dataset 4, where all data points were captured by the model with the exception of year 2 in the low intensity group. In all years however, matrix D captured the 95 % CIs of all observed data points.

**Table 6:** Predicted mean prevalence by matrix A for dataset 4 (longitudinal Mali data)

	Low intensity (predicted mean prevalence and 95 % CI)		Moderate intensity (predicted mean prevalence and 95 % CI)		High intensity (predicted mean prevalence and 95 % CI)		Overall prevalence (predicted mean prevalence and 95 % CI)	
Matrix	Year 1	Year 2	Year 1	Year 2	Year 1	Year 2	Year 1	Year 2
**Observed prevalence dataset 4**	0.113 (0.094–0.135)	0.122 (0.102–0.145)	0.052 (0.040–0.069)	0.036 (0.025–0.050)	0.023 (0.015–0.036)	0.008 (0.004–0.016)	0.188 (0.164–0.215)	0.165 (0.142–0.191)
**Matrix A** Full dataset	0.112 (0.095–0.129)	**0.096** **(0.079–0.115**)	0.049 (0.039–0.059)	0.035 (0.025–0.046)	0.027 (0.020–0.035)	**0.018** **(0.011–0.027)**	0.188 (0.169–0.207)	0.149 (0.126–0.174)

**Bold** = observed point prevalence values fell outside of the predicted boundaries

## Discussion

The primary aim of this study was to develop a simple quantitative tool to help programme managers to monitor and evaluate the ongoing progress of their schistosomiasis disease control interventions and whether they are meeting their targets. For this, we parameterized and validated Markov models using an extensive longitudinal dataset of *S. mansoni* infection in Ugandan children treated yearly with PZQ. Additionally, in order to test the robustness of the model predictions in a completely different setting, we compared model predictions against data from comparable school-aged children from the national control programme in Mali. Our focus was on the ability of the models to capture the observed point prevalence values, as a conservative approach to model assessment. It is anticipated that programme managers will be able to use their own baseline and year 1 data to predict changes in infection prevalence in subsequent years of the same programme, as this is the scenario where the model performed best.

Our study therefore demonstrated that this Markov modelling approach is useful when making (relatively short-term) predictions on infection trends with large datasets from which a subset has been used to parameterize the model (as seen by matrix A *vs* dataset 1 and matrix D *vs* dataset 4). Additionally, it is useful when completely independent data from another country have been used to parameterize the model and when predicting cross-sectional data. These results are particularly noteworthy since the vast majority of sentinel site survey data tend to be cross-sectional in design given inherent logistical and financial advantages. Matrices A and B performed similarly (with matrix A predicting changes in prevalence correctly within the 95 % PI range at more follow-up times in each infection intensity group than any other matrix variant), showing that the models performed similarly, whether TPs developed from baseline to year 1 data (matrix A) or from year 1 to year 2 (matrix B) were used to parameterize the model. It is important to test the performance of the model on a completely different country as this is 1 scenario for which a programme manager may use this model, and for these reasons data from Mali (dataset 4) were used to both separately test the model with Ugandan TPs (matrix A) and parameterize the model (baseline and year 1 data for matrix D). The model was able to predict a large majority of data points within the estimated 95 % PIs, in both cases: matrix A predicted all but 2 data points within the 95 % PIs (but captured the 95 % CIs around the data) for Mali dataset 4, and matrix D performed similarly to matrices A and B when predicting dataset 1. Conversely, matrix C (using data from selected districts in Uganda) performed least well, with only 16 of the 36 estimates in this study capturing the observed point prevalence values within the 95 % PIs. However, it is not possible to determine how the trends would continue without further data; therefore, this study is limited to the data we had available.

We conclude that, in its current form, the model is a useful additional tool for programme managers, provided they have the data available for the parameterisation of the model to the local setting, and is particularly useful for the interpretation of data from low and high infection intensity areas where all of the models performed best. This is ideal for programmes preparing to move from control of morbidity to interruption of transmission and elimination of infection (more feasible in low infection intensity areas) or to elimination of schistosomiasis as a public health problem (more severe in high infection intensity areas). Availability of longitudinal follow-up data is not essential, provided the sample size is large (as in this study) for cross-sectional annual data; however, longitudinal data are required to calculate the TPs. The use of data from Mali for parameterization (matrix D) illustrated that the model could, with some caution, be considered useful for predicting prevalence changes in Uganda, but more data would be required from other countries to test this further.

These models are aimed to be a tool to aid decisions and stimulate further investigation when needed rather than be used as a precise prediction of likely impact. Therefore, it is hoped that this heuristic technique may be useful for programme managers as a quick and simple means of assessing the progress of programmes. However, as seen by the results concerning dataset 4 (Mali longitudinal cohort), it is important to interpret the data for all 4 infection intensity groups together, since a large observed increase in the low infection intensity group compared to model outputs, may likely be linked to a corresponding decrease in the proportion of the heavier infection intensity groups. The precise change in infection patterns following treatment will depend on a multitude of factors related to programmatic design and performance. These will include therapeutic coverage and treatment adherence, which in turn will be related to other programmatic variables, such as the performance of the drug distribution teams, the accuracy of census data, and the effectiveness of social mobilization techniques, among others. Identifying the respective impact of each of these factors is beyond the scope of this paper.

Despite its advantages, the limitations of the Markov approach must be understood if it is to constitute a useful tool by programme managers. The model employed in this study is referred to as a time-homogenous Markov process [28], which assumes that the TPs remain constant through time. It is also assumed that they are invariant with respect to setting (endemicity, geographic location etc.) and host age group. This is not likely to hold for long-term projections as interventions (in this case MDA) are likely to have an impact on the transmission environment. For these reasons, such models may indicate ‘abnormalities’ in the observed datasets as a result of inevitable or expected changes over time, therefore the usefulness of the approach resides in its value as an additional tool for monitoring and evaluation rather than the definitive tool for this purpose. The data used to validate and test the models are primarily from school-aged children since most schistosomiasis interventions focus on this age group, who tend to harbour the highest burden of infection [29–35]. Therefore, the models do not consider the broader impact of MDA on the entire community *via* the indirect (herd) effects on transmission that result from reducing the force of infection [13]. Moreover, the method also implies that the same intervention is used each year using the same treatment schedule, not accounting for complementary interventions that may be implemented, such as those relating to sanitation or education, increase in public awareness that may accompany the progression of a control programme, or changes in the frequency and/or coverage of MDA. The model is based on a closed system and, therefore, assumes no population migration or extraneous introduction of new infections. This is an important limitation for mobile communities that may comprise so-called super-spreading individuals (such as fishermen or bicycle washers) who contribute disproportionately to community-wide transmission and who may be more likely to miss treatment. However, this is also a general limitation of most helminth transmission models, which rarely consider the spatial aspects of transmission.

With these limitations in mind, this study demonstrates that using constant TPs from the same dataset or from different datasets provides a satisfactory prediction of data (and their uncertainty) on the overall prevalence and the prevalence of high, moderate and light infections for up to 3 follow-up years. This method could also be extended to *S. haematobium*, adapting the model to the different WHO intensity classes for this species (defined as 1–50 eggs/10 ml of urine as light intensity and > 50 eggs/10 ml of urine as heavy intensity, with no moderate intensity group) [9, 19] as well as to *S. japonicum*. In this case, the transmission dynamics among multiple definitive hosts would potentially pose less of a problem to this modelling approach when compared to other models that do not take into account the zoonotic reservoir, as the TPs calculated from the initial data would include all of the transmission-related processes occurring between the 2 time points [36–38]. This study could also be expanded further by comparing different TPs obtained from other datasets. In addition, the models could be adapted to make longer-term predictions (since the present study is focussed on short-term changes of 1–3 years post-baseline due to the stationary TP limitation), using datasets spanning longer periods and incorporating MDA coverage information. These extensions could, in principle, be captured using multiple TPs based on existing data of varying treatment coverage, or the possibility of having dynamic TPs that change with time or are simply updated as new data become available (developing new TPs from the more recent followed cohort). The use of year 1 to year 2 TPs in this study illustrated the potential for updating TPs as the programme progresses to estimate changes in subsequent years. This would overcome the constraints imposed by using baseline and year 1 data only, for projecting over long running programmes.

## Conclusions

We developed and refined a Markov model to capture changes in the prevalence of infection intensity categories for *S. mansoni* infection over multiple rounds of MDA with PZQ. We parameterized our model using 2-year (2 consecutive time points) longitudinal data from Uganda and from Mali, using it to make longer-term projections against different variations of the datasets. The results from this study show that this is not only a promising instrument for programmes in their early years of implementation as a complementary M&E tool, but also a useful quantitative approach for making short-term projections of prevalence trends under interventions. With the ambitious WHO 2020 goals on the horizon, there is a need to look beyond maintaining control of schistosomiasis and shift focus to eliminating this debilitating disease. The global research community needs to develop practical tools to help programmes to achieve these goals. The Markov model has already produced encouraging results with existing programmatic data. With the push towards the elimination of schistosomiasis as a public health problem by 2020, these findings come at a key time in the field of NTD modelling for programme managers and policy makers.

## Additional file

Additional file 1: Markov model equations, model parameters and additional tables and figures. **Text S1**. Markov model formulae. **Table S1**. Definition of parameters for the Markov Model. **Table S2**. Uganda subset information for dataset 2 and matrix C. **Figure S1**. Results from applying transition probability (TP) matrices B and C on dataset 1 (full longitudinal Uganda data set). **Figure S2**. Results from applying TP matrices A–C on dataset 2 (selected Ugandan districts). **Figure S3**. Results from applying TP matrices B and C on dataset 3 (cross-sectional Ugandan dataset). **Figure S4**. Results from applying TP matrix D (baseline and year 1 Mali data for TPs) on dataset 4 (longitudinal Mali dataset). (DOC 6719 kb)

## Abbreviations

CI: Confidence interval
CS: Conditional state
DALY: Disability-adjusted life year
EPG: Eggs per gram of faeces
M&E: Monitoring and evaluation
MDA: Mass drug administration
MTP: Markov Transition Probability
NGO: Non-governmental organization
NTD: Neglected tropical disease
PC: Preventive chemotherapy
PI: Prediction interval
PI: Prediction interval
PZQ: Praziquantel
SAC: School-aged children
SCI: Schistosomiasis Control Initiative
SSA: Sub-Saharan Africa
STH: Soil-transmitted helminth
TP: Transition probability
WHO: World Health Organization
