# Supplementary material for: Development and evaluation of a Markov model to predict changes in schistosomiasis prevalence in response to praziquantel treatment: a case study of Schistosoma mansoni in Uganda and Mali
Source: Parasit Vectors. 2016 Oct 12;9:543. doi: 10.1186/s13071-016-1824-7 (PMC5059905; doi:10.1186/s13071-016-1824-7)

*Additional File 1*

**Markov model equations, model parameters and additional tables and figures**

**Text S1. Markov model formulae**

The following equations have been adapted from Montresor A, et al. (2013), which calculate the transition probabilities between two time points (*t* and *t_t+1_*) using a transition probability matrix:


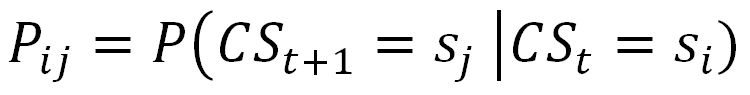
 (1)

where *P_ij_* is defined as the probability of moving from state *i* to state *j* after one follow up round (which is 1 year for this study), and assumed to be time-homogeneous and dependent only on *CS_t_* (Eqn. 1).

The probability distribution of the initial state is known and represented by an *s* x *s* matrix (i.e. the TP matrix *P*), where *s* represents the discrete conditional states (Eqn. 2).


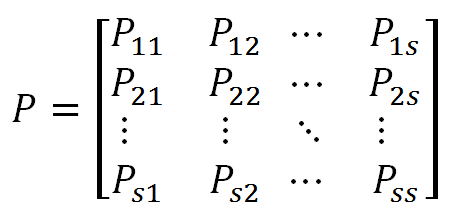
 (2)

Where for all *i,j*:


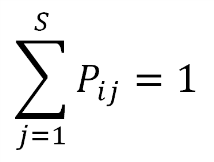


and (3)


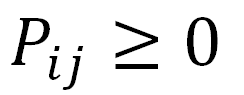


The probability of moving from one conditional state to another can then be calculated using Eqn. 4.


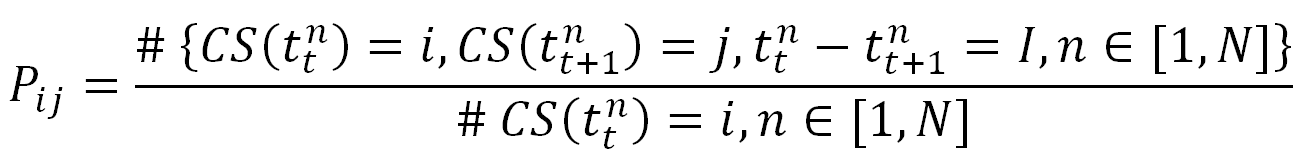
 (4)

**Table S1.** Definition of parameters for the Markov Model

| **Symbol** | **Definition** | **Description** |
| --- | --- | --- |
| *P­_ij_* | Transition probability | Probability of moving from state *i* to state *j* after one follow up round |
| *CS* | Conditional state | CS is used to describe the state space |
| 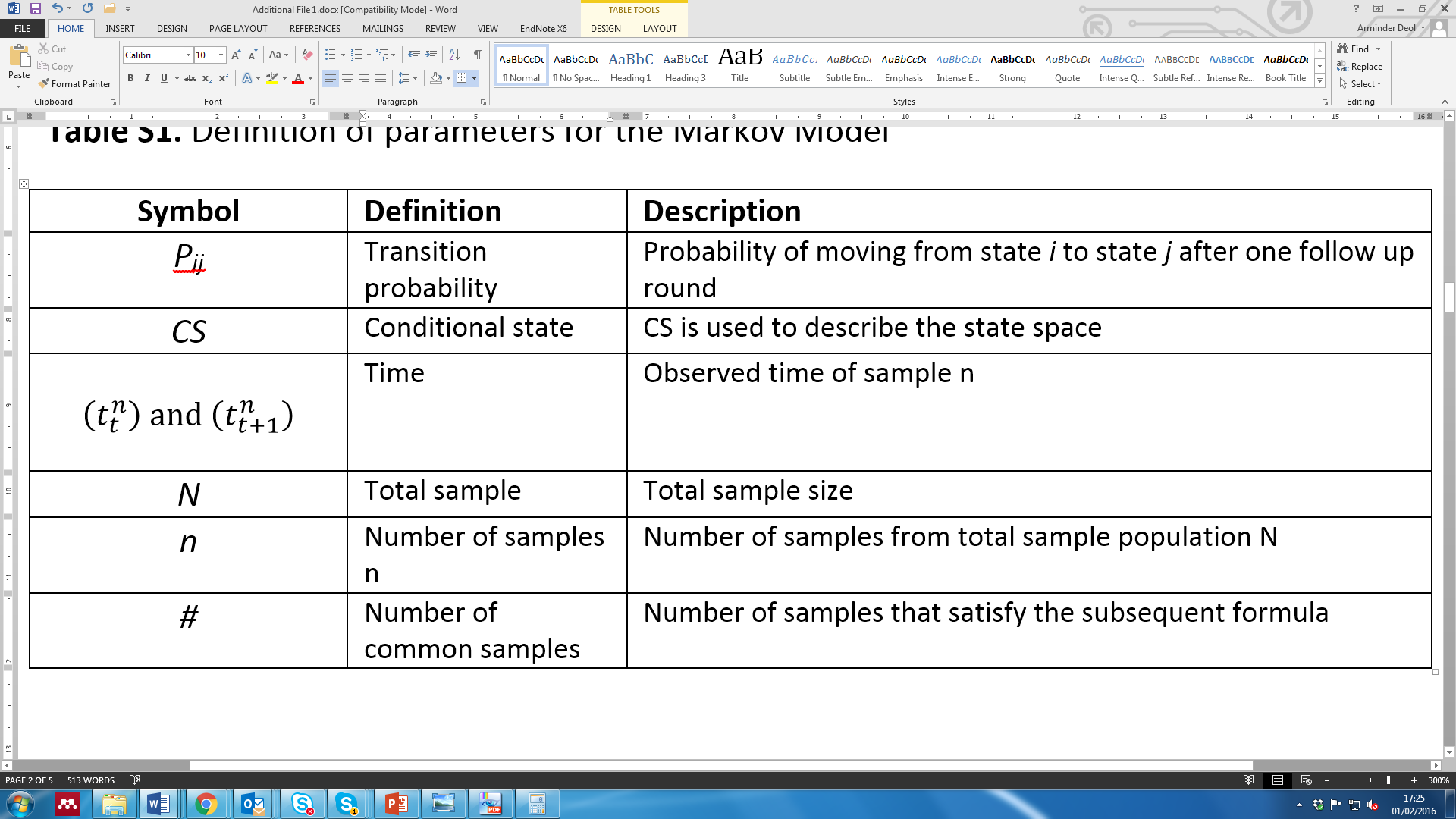 | Time | Observed time of sample n |
| *N* | Total sample | Total sample size |
| *n* | Number of samples n | Number of samples from total sample population N |
| *#* | Number of common samples | Number of samples that satisfy the subsequent formula |

**Table S2.** Uganda data subset information for dataset 2 and matrix C

| **District** | **Sample size (all years followed)** | **Sample size followed from baseline- year 1 (matrix C)** | **Intensity groups in each district** |
| --- | --- | --- | --- |
| Moyo | 142 | 217 | Low |
| Mayuge | 85 | - | Low/High |
| Masindi | 69 | 128 | Med/High |
| Bugiri | 110 | - | Low/High |
| Busia | 142 | 204 | Low/Med |
| Hoima | 99 | - | Low/Med/High |
| Nebbi | 110 | - | Low/Med/High |
|  |  |  |  |
| **Total** | **757** | **549** |  |
| **Total not used for TPs (dataset 2)** | **404** | **-** |  |

**Figure S1.** Results from applying transition probability (TP) matrices B and C on dataset 1(full longitudinal Uganda data set)

Figure S1a Matrix B (using year 1 and year 2 Ugandan data for TPs) and dataset 1


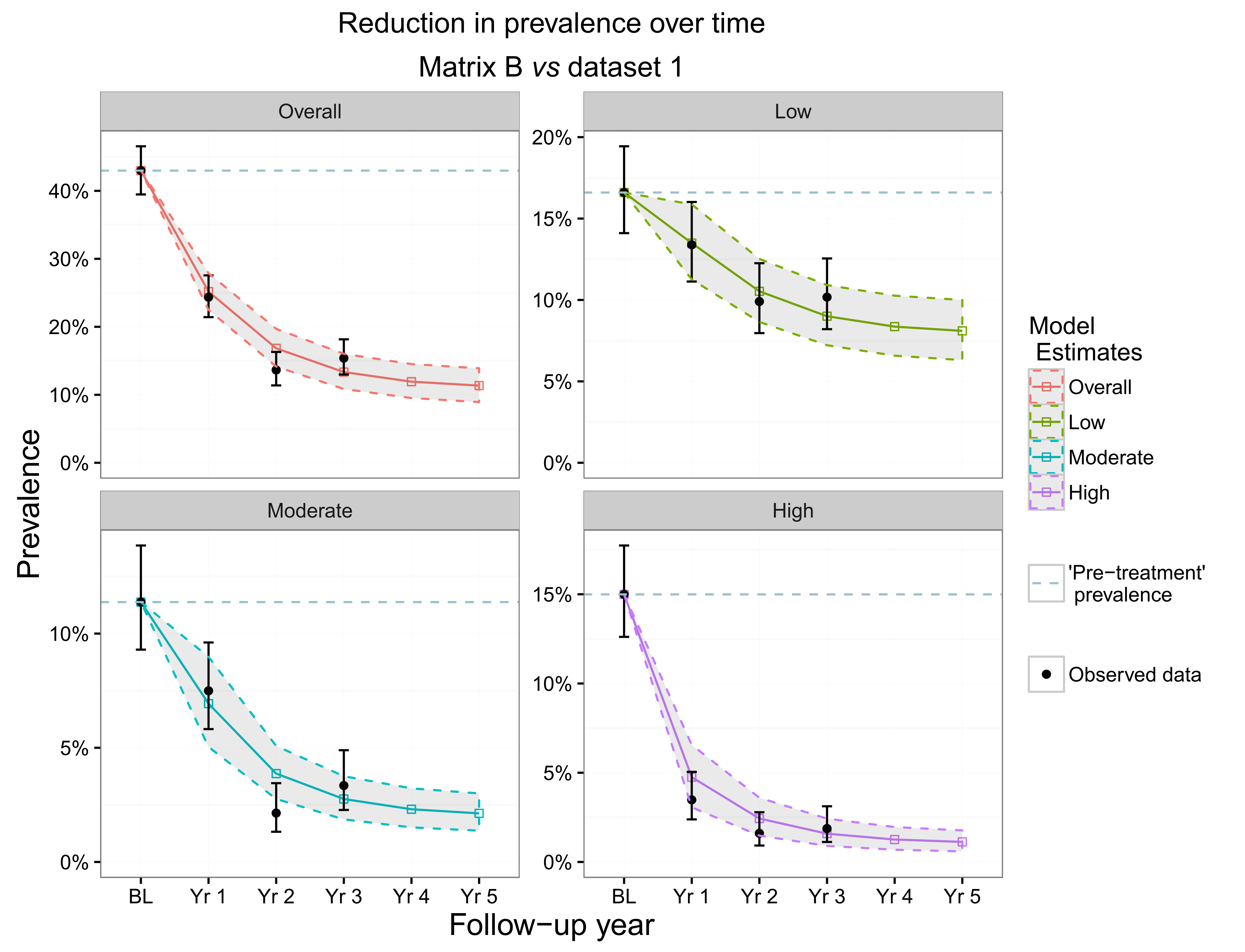


Figure S1b Matrix C (selected Ugandan districts for TPs) and dataset 1

**
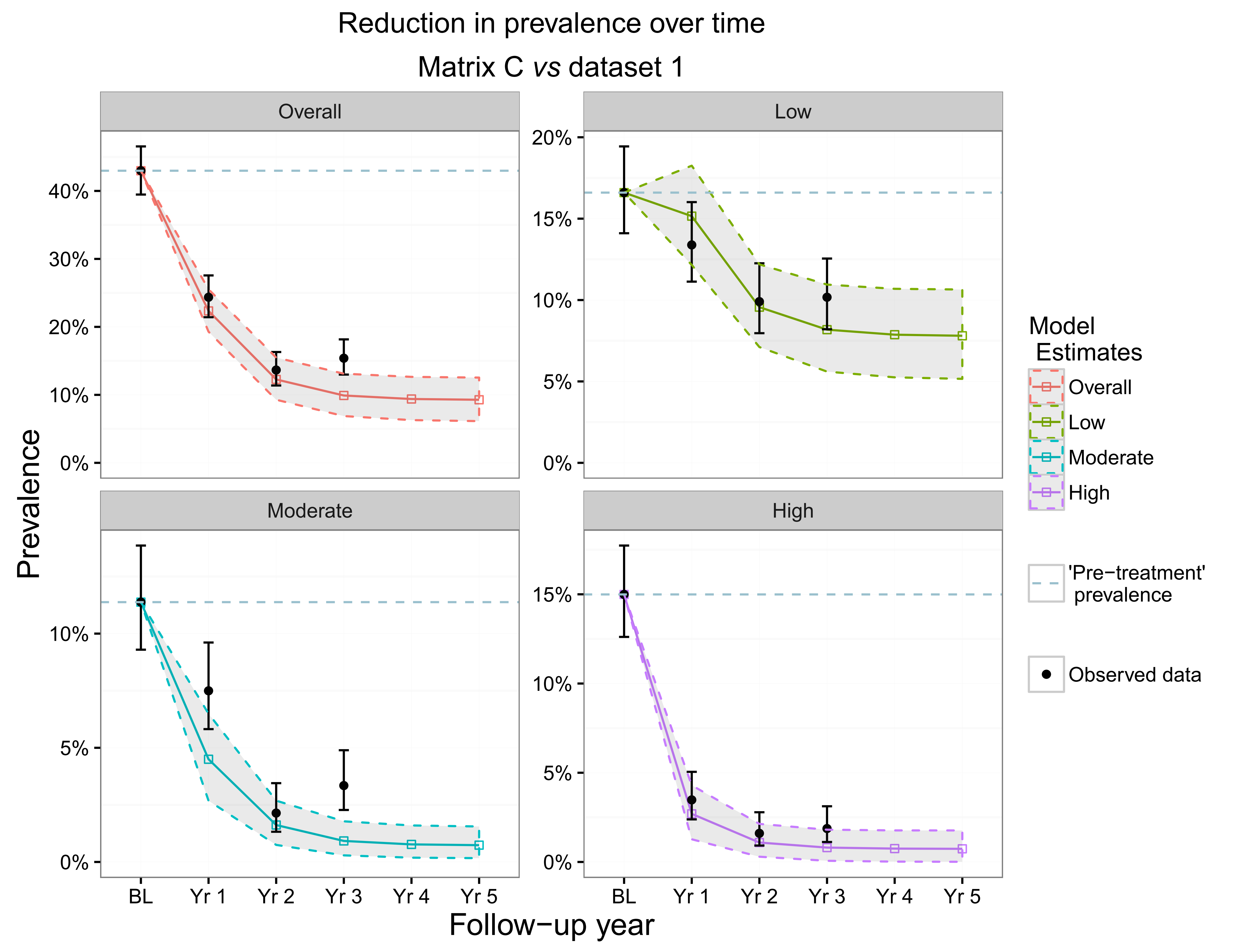
**

**Figure S2.** Results from applying TP matrices A-C on dataset 2 (selected Ugandan districts)

Figure S2a Matrix A (full Ugandan data for TPs) and dataset 2


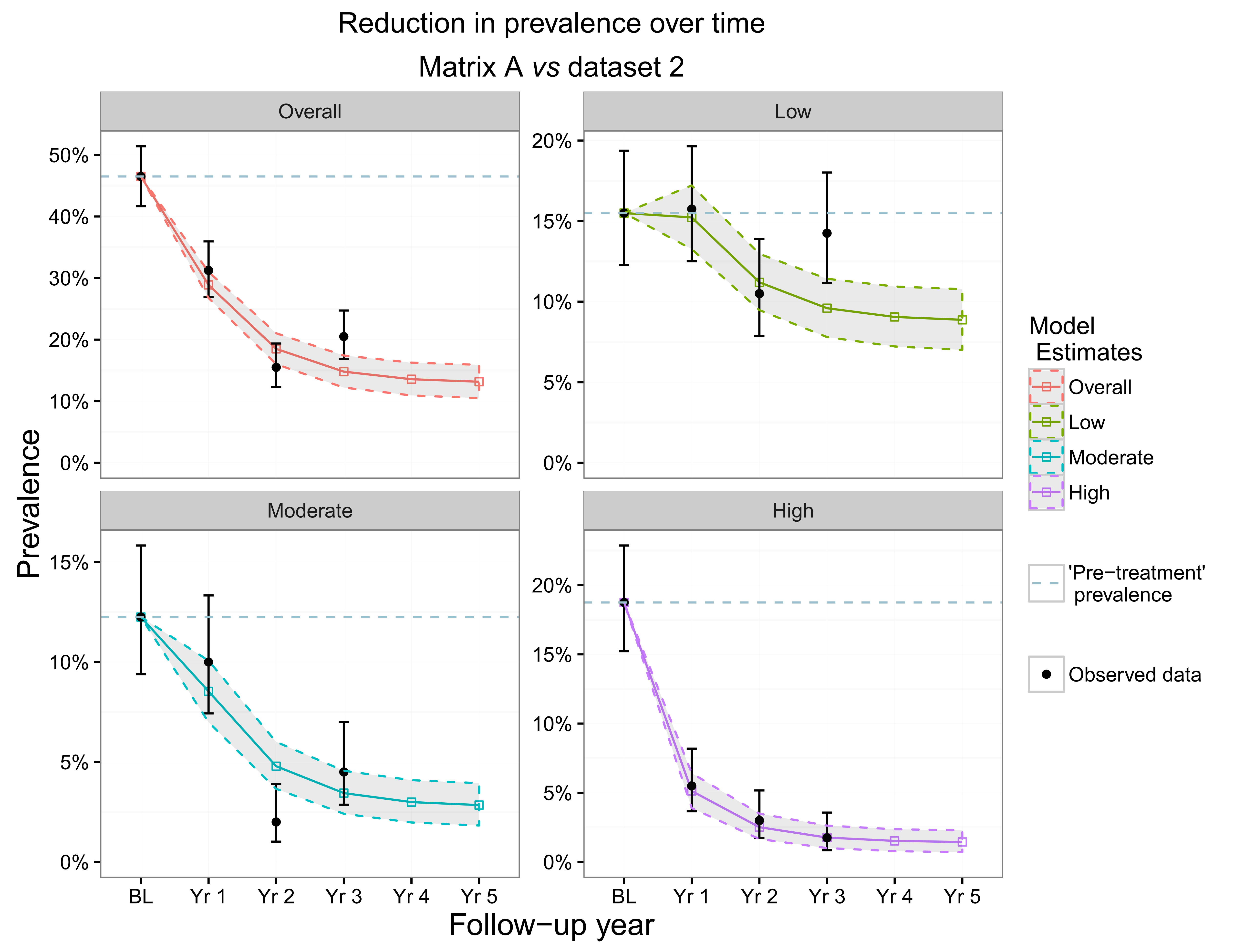


Figure S2b Matrix B and dataset 2


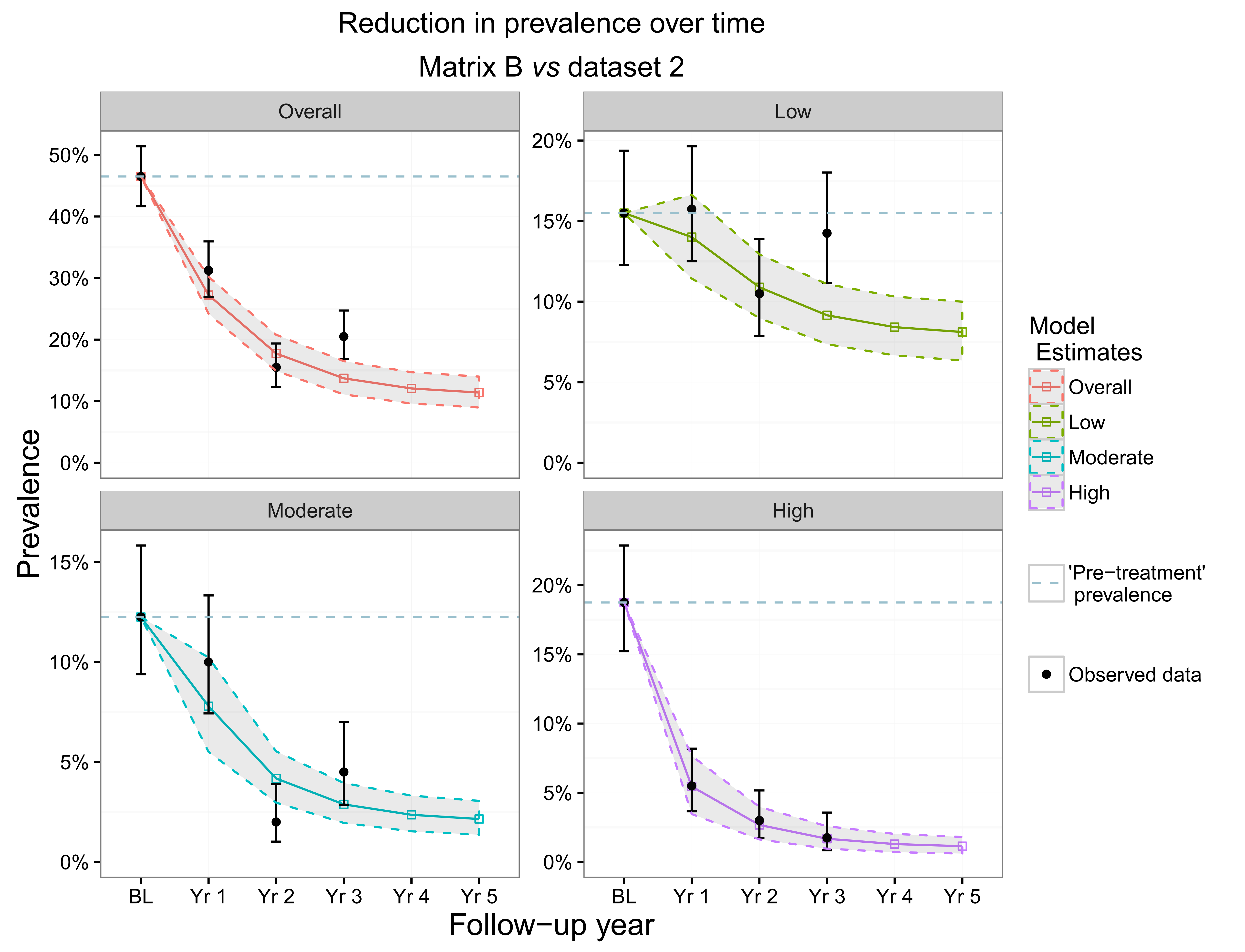


Figure S2c Matrix C and dataset 2


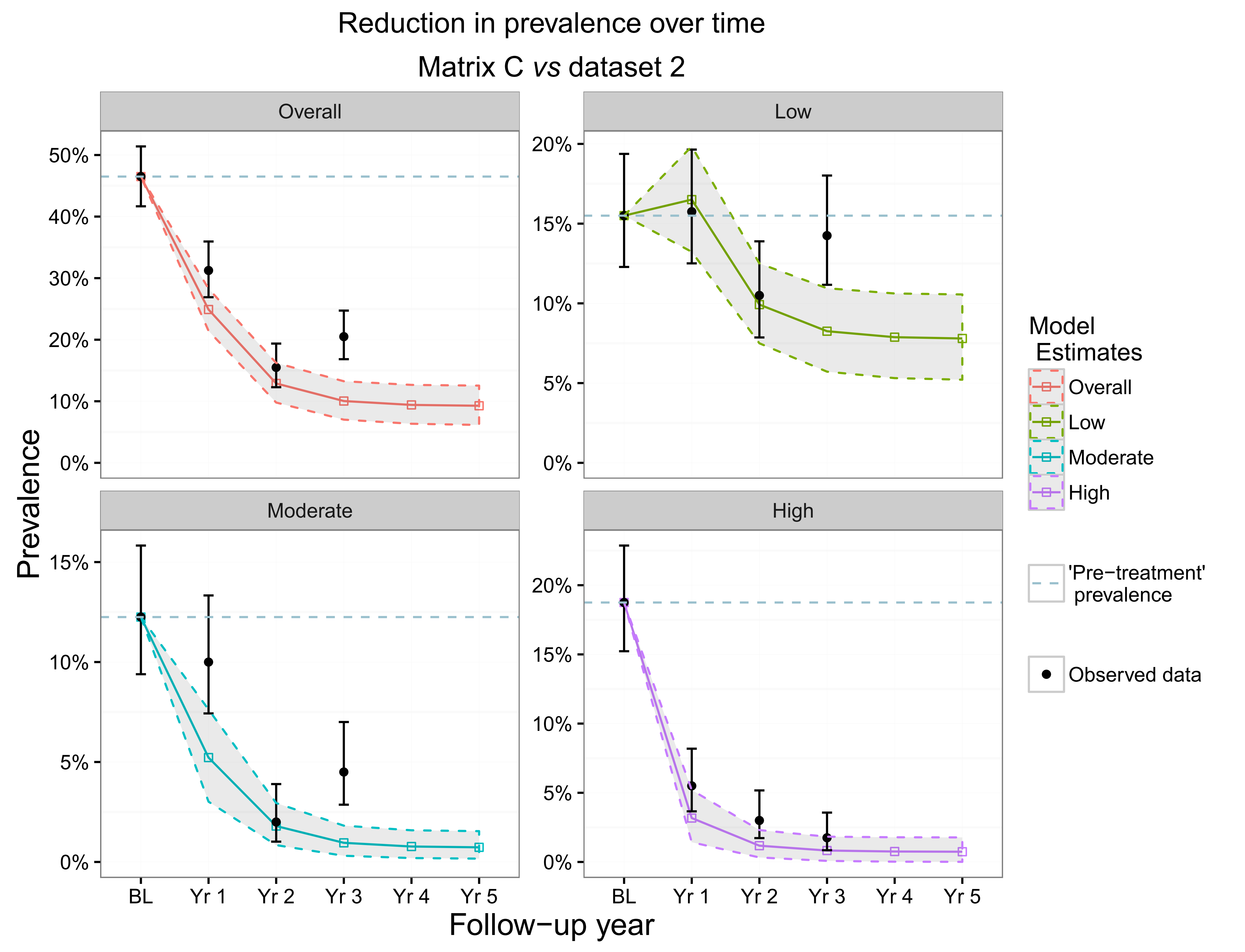


**Figure S3.** Results from applying TP matrices B and C on dataset 3 (cross-sectional Ugandan dataset)

Figure S3a Matrix B and dataset 3


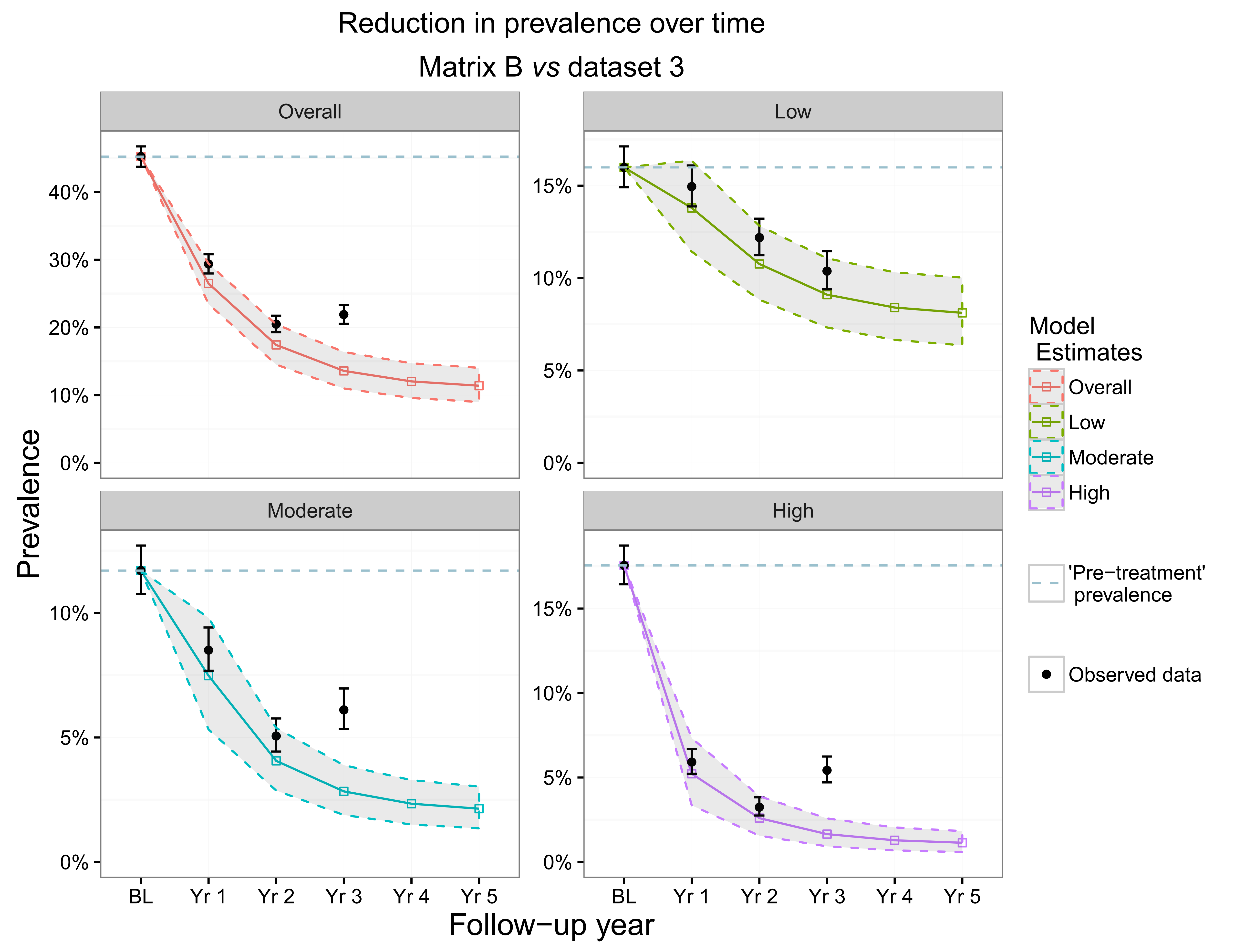


Figure S3b Matrix C and dataset 3


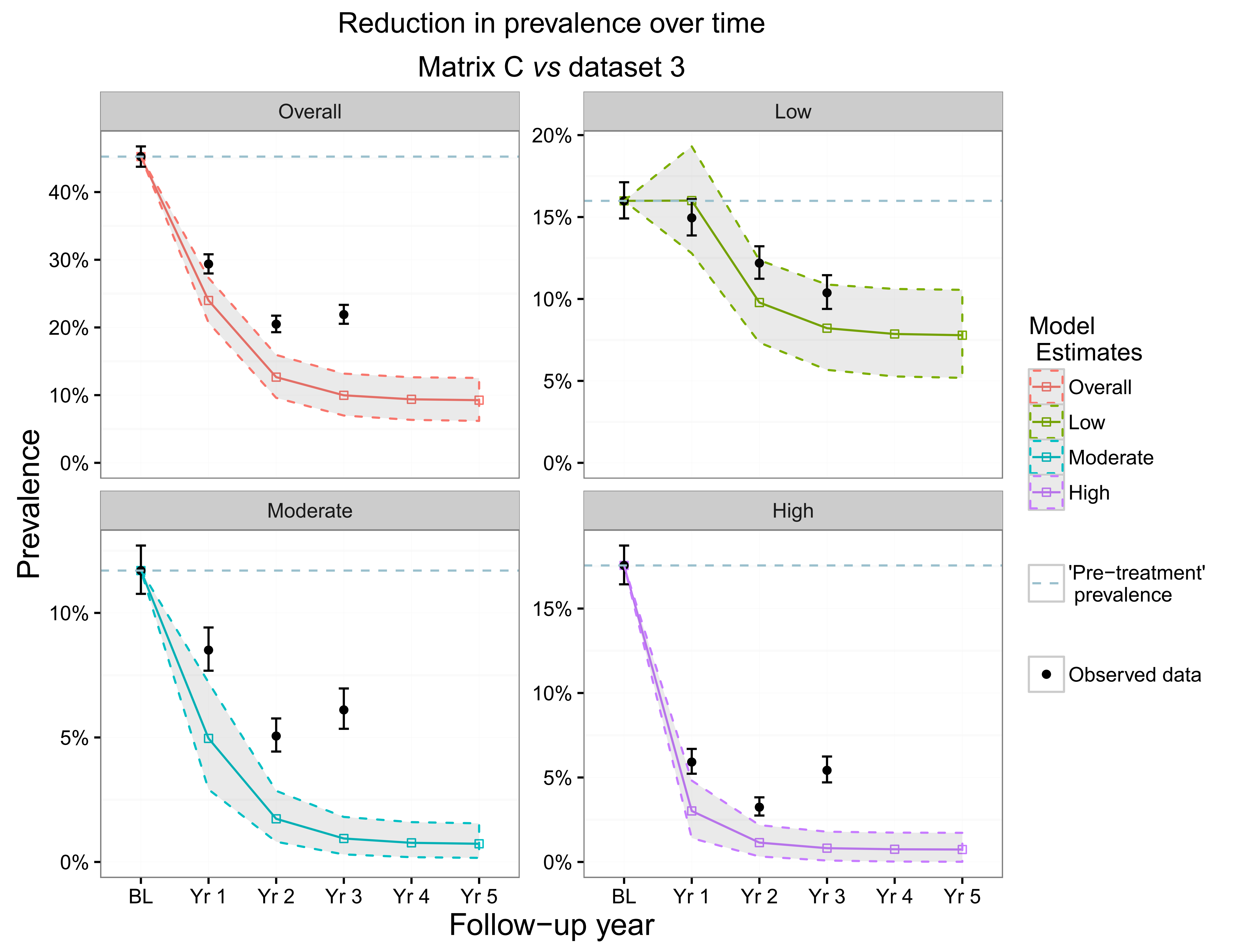


**Figure S4.** Results from applying TP matrix D (baseline and year 1 Mali data for TPs) on dataset 4 (longitudinal Mali dataset)


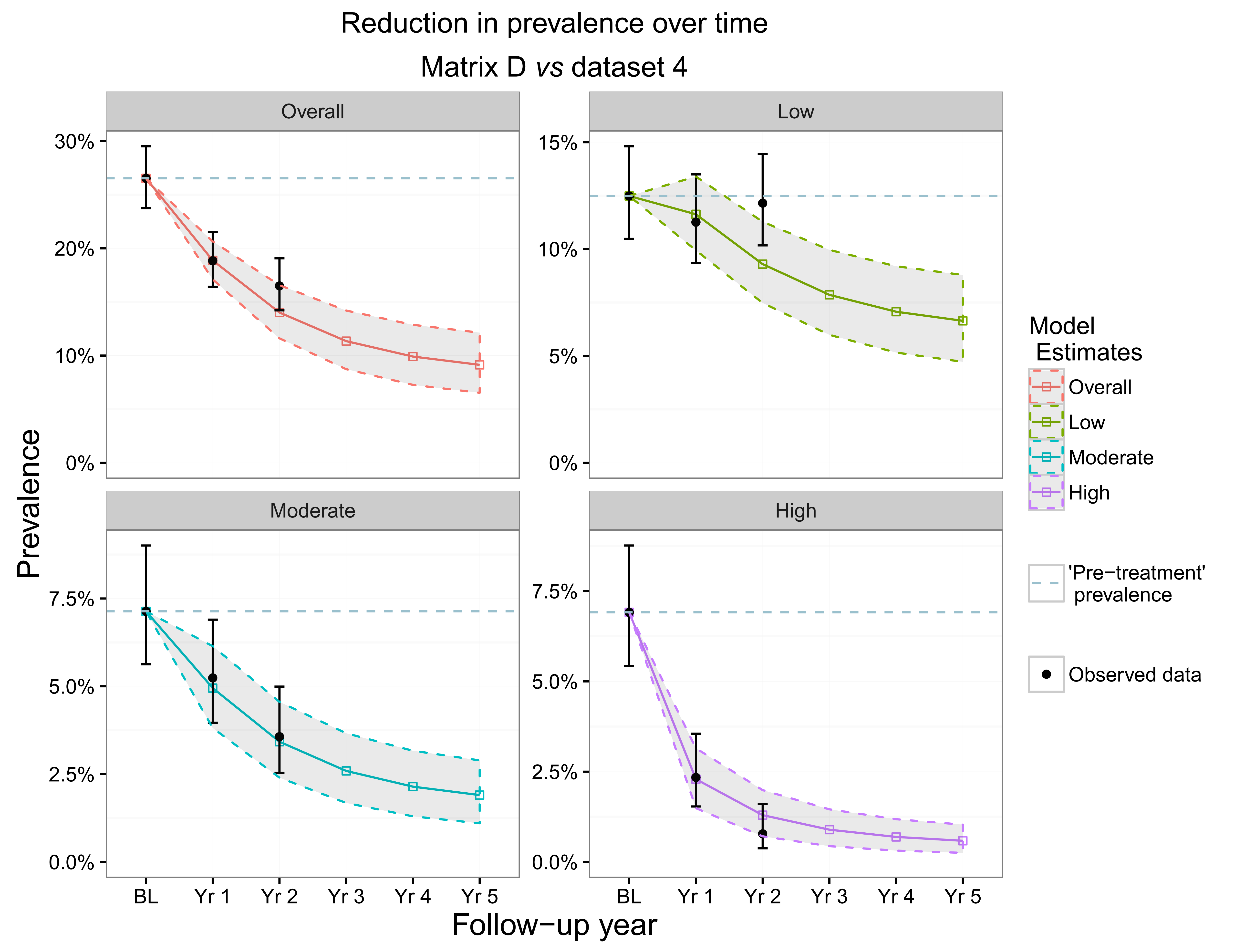

Supplement: Additional file 1: — Markov model equations, model parameters and additional tables and figures. Text S1. Markov model formulae. Table S1. Definition of parameters for the Markov Model. Table S2. Uganda subset information for dataset 2 and matrix C. Figure S1. Results from applying transition probability (TP) matrices B and C on dataset 1 (full longitudinal Uganda data set). Figure S2. Results from applying TP matrices A–C on dataset 2 (selected Ugandan districts). Figure S3. Results from applying TP matrices B and C on dataset 3 (cross-sectional Ugandan dataset). Figure S4. Results from applying TP matrix D (baseline and year 1 Mali data for TPs) on dataset 4 (longitudinal Mali dataset). (DOC 6719 kb) [file 13071_2016_1824_MOESM1_ESM.doc]
